# Effectiveness of anti-PD-1/PD-L1 antibodies in urothelial carcinoma patients with different PD-L1 expression levels: a meta-analysis

**DOI:** 10.18632/oncotarget.24249

**Published:** 2018-01-13

**Authors:** Junqi Liu, Chuanfeng Zhang, Jiegang Hu, Qing Tian, Xin Wang, Hao Gu, Song Zhang, Di Zhao, Ruitai Fan

**Affiliations:** ^1^ Department of Radiotherapy, The First Affiliated Hospital of Zhengzhou University, 450000 Zhengzhou, Henan, China; ^2^ Endocrinology Department, The First Affiliated Hospital of Zhengzhou University, 450000 Zhengzhou, Henan, China

**Keywords:** programmed death-1 (PD-1), programmed death-ligand 1 (PD-L1), urothelial carcinoma, meta-analysis

## Abstract

**Background:**

Urothelial carcinoma ranks the ninth among malignant cancers. We conducted this study to identify which patients could benefit more from the treatment of programmed death-1 (PD–1)/programmed death-ligand1 (PD–L1) inhibitors.

**Materials and Methods:**

We performed literature searches, combined data from qualified literature and performed comparative analyses on the effectiveness of anti-PD–1/PD–L1 antibodies in patients with different PD–L1 expression levels.

**Results:**

We divided patients into three groups according to the percentages of PD–L1-positive cells, namely the low- PD-L1 (PD-L1 < 1%), the medium-PD-L1 (PD-L1 ≥ 1 and < 5%) and the high–PD–L1 (PD-L1 ≥ 5%) groups. We found that the high-PD-L1 group responded significantly better than other groups (P = 0.0003, ORs = 0.45, 95%CI: 0.29-071; P = 0.0009, ORs = 0.43, 95%CI: 0.25-0.73, for low-PD-L1 and medium-PD-L1 groups, respectively), while the latter two groups responded similarly (P = 0.90, ORs = 1.06, 95%CI: 0.62-1.83) to both PD–1 and PD–L1 inhibitors. Furthermore, we found that the medium-PD–L1 and high-PD–L1 groups responded similarly to PD-1/ PD-L1 inhibitors (P = 0.65, ORs = 1.11, 95%CI: 0.69–1.77), while the low-PD–L1 group responded better to PD-1 inhibitors than PD-L1 inhibitors (P = 0.046, ORs = 1.92, 95%CI: 0.98–3.89).

**Conclusions:**

Our results suggest that PD–L1 positive patients should be defined as those with ≥ 5% or greaterPD-L1-positive cells. PD-1 antibodies performed better only in the low-group patients, likely because they could block the interactions of PD–1 with both PD–L1 and PD–L2.

## INTRODUCTION

The incidence of urothelial carcinoma has become the ninth of all malignant tumors and the first in urinary tract cancers [1]. It leads to the death of more than 165,000 people every year all over the world [2]. Urothelial cancer can be divided into two groups: upper tract urothelial carcinoma and urinary bladder carcinoma. Both of the them occur more often in males than in females [3]. Platinum-based chemotherapy has been the first-line choice in advanced and metastatic urothelial cancer, but often has very poor prognosis [4]. It is thus urgent that new therapeutic options should be introduced for patients with urothelial cancer.

Tumor microenvironment plays an important role in tumor immunity [5]. Programmed death-ligand1 (PD-L1) is found to be expressed in human tumor-associated antigen presenting cells, and can suppress the immune system through its receptor programmed death-1 (PD-1) and lead to immune escape of tumor cells [6, 7]. So far a few PD-1 and PD-L1 antibodies are commercially available, including PD-1 antibodies nivolumab and pembrolizumab, and PD-L1 antibodies avelumab, atezolizumab and durvalumab. These antibodies have showed very good antitumor effects on advanced and metastatic cancers, including melanoma [8], non small cell lung cancer (NSCLC) [9], squamous cell carcinoma of the head and neck [10], urothelial carcinoma [11] and renal cell carcinoma [12]. Some clinic trials have shown that the use of PD-1/PD-L1 antibodies resulted in better clinical outcomes than platinum-based chemotherapy [13–15].

It has been shown in many clinical trials that cancer patients with higher expression of PD-L1 received better antitumor effect to PD-1/PD-L1 inhibitors [8, 11]. Similar results have been observed in urothelial cancer [11, 16]. However, in recent studies there have been some seemingly different observations. For example, some researchers found that patients with ≥ 1% PD-L1-positive cells responded better to PD-1/PD-L1 antibodies; however, some other studies found similar effects only in patients with ≥ 5% PD-L1-positive cells [17]. Although the limited numbers of patients in individual studies could be a reasonable explanation to these different results, they did raise the critical question regarding the threshold for distinguishing PD-1/PD-L1 positive patients from negative ones [18]. In addition, it is still unclear for oncologists that which antibodies (i.e. PD-1 or PD-L1 antibodies) work better for patients with urothelial cancer. We therefore performed a meta-analysis to address the following two questions. First, what threshold should be used for distinguishing PD-1/PD-L1 positive patients from negative ones? Second, which antibodies (i.e. PD-1 or PD-L1 antibodies) work better for patients with urothelial cancer?

## RESULTS

### Six eligible studies were included for analysis

**Figure 1:**
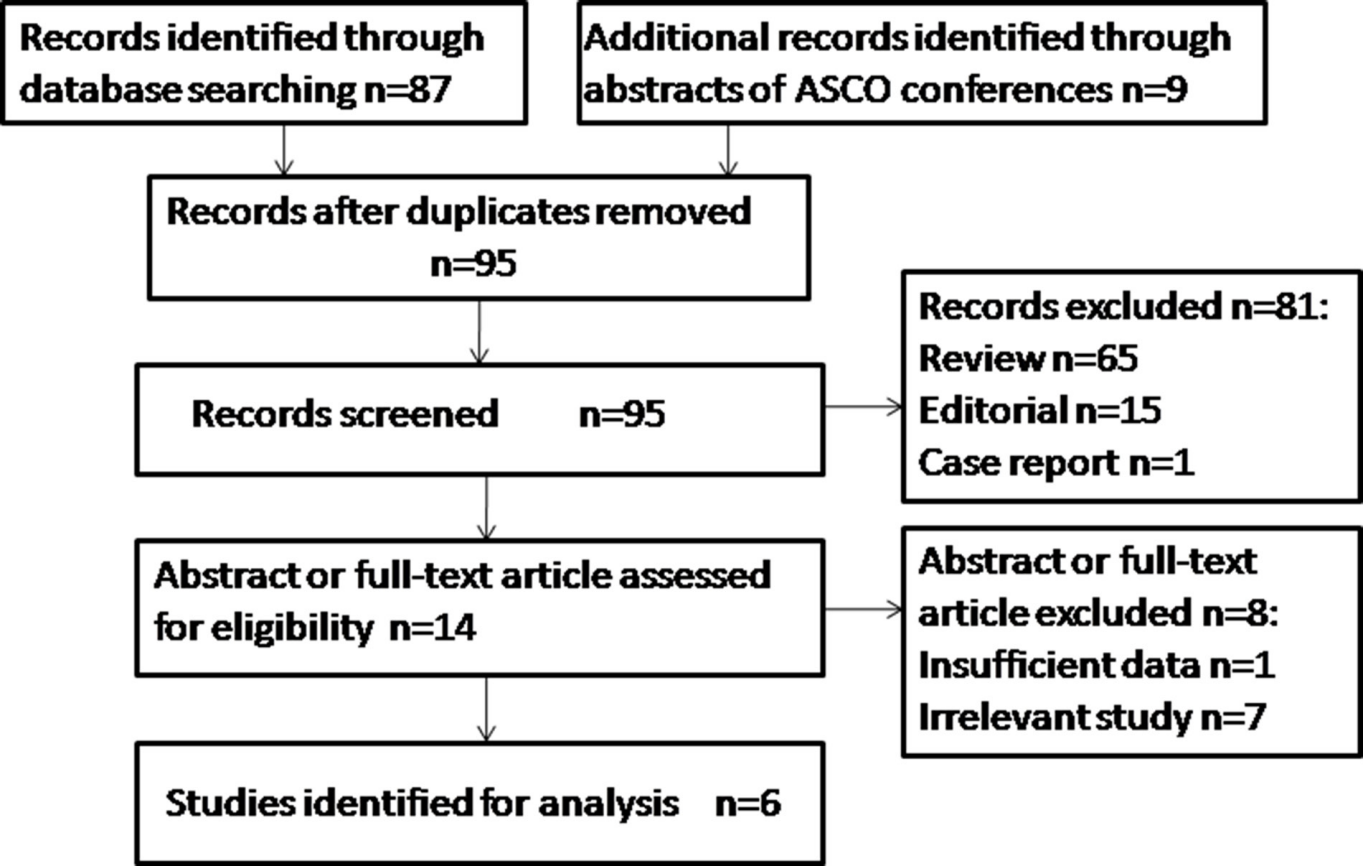
Work flow for study selection In total 95 articles were retrieved from public databases. Six articles involving 828 participants were kept for further analysis.

We retrieved in total 95 articles from public databases using the above-mentioned keywords. After reading abstracts and full texts, we kept six articles for further analysis; in total 828 participants were tested in the six studies. Please consult Figure 1 for the overall workflow of the literature selection, the criteria of the selection and the numbers of articles remained/excluded at each step.

Three out of the six studies evaluated PD-1 antibodies, of which two evaluated nivolumab [19, 20] and the other evaluated pembrolizumab [21]. Three studies evaluated PD-L1 antibodies, of which two evaluated atezolizumab [11, 16] and the other evaluated durvalumab [22]. All of the studies were single-arm trials (see Table 1 for details).

**Table 1:** Characteristic of eligible literatures

Trials	Phase	Trial name	Antibody	PD-L1 expression	ORR	Median PFS	Media OS
Sharma.et.al.2016 [20]	I/II	CheckMate 032	Nivolumab ^[1]^	All	24.4% (95%CI 15.3–35.4)(19/78)	2.8 months	9.7 months
				PD-L1 < 1%	26% (95%CI 14-42)(11/42)	2.8 months	9.9 months
				PD-L1 ≥ 1%	24% (95%CI 9-45)(6/25)	5.5 months	16.2 months
Sharma.et.al.2017 [19]	II	CheckMate 275	Nivolumab ^[1]^	All	19.6% (95%CI 15.0–24.9)(52/265)	—	8.74 months
				PD-L1 < 1%	16.1% (95% CI 10.5-23.1)(23/143)	—	5.95 months
				PD-L1 ≥ 1%	23.8% (95%CI 16.5-32.3)(29/122)	—	11.30 months
				PD-L1 < 5%	23.8% (29/184)	—	—
				PD-L1 ≥5%	28.4% (95%CI 18.9-39.5)(23/81)	—	—
Plimack.et.al.2017 [21]	Ib	KEYNOTE-012	Pembrolizumab ^[1]^	All	26% (95%CI 11-46)(7/27)	2 months	13 months
				PD-L1 < 1%	0 (95%CI 0−60)(0/4)	—	—
				PD-L1 ≥ 1%	24% (95%CI 8−47)(5/21)	—	—
Rosenberg.et.al.2016 [11]	II	NCT02108652	Atezolizumab ^[2]^	All	15% (95%CI 11-19)(45/310)	2.1 months	7.9 months
				IC0 < 1%	8% (95%CI 3–15)(8/103)	—	—
				IC1/2/3 ≥ 1%	18% (95%CI 13-24)(37/207)	2.1 months	8.8 months
				IC1 ≥ 1% but < 5%	10% (95%CI 5–18)(11/107)	—	—
				IC2/3 ≥ 5%	26% (95%CI 18-36)(26/100)	2.1 months	11.4 months
Balar.et.al.2017 [16]	II	NCT02108652	Atezolizumab ^[2]^	All	23% (95%CI 16-31)(27/119)	2.7 months	15.9 months
				IC0 < 1%	21% (95%CI 9–36)(8/39)	2.6 months	—
				IC1/2/3 ≥ 1%	24% (95%CI 15–35)(19/80)	—	—
				IC1 ≥ 1% but < 5%	21% (95%CI 10–35)(10/48)	2.1 months	—
				IC0/1 < 5%	—	—	19.1 months
				IC2/3 ≥ 5%	28% (95%CI 14–47)(9/32)	4.1 months	12.3 months
Massard.et.al.2016 [22]	I/II	NCT01693562	Durvalumab ^[2]^	All	31% (95%CI 17.6-47.1)(13/42)	—	—
				PD-L1 < 1%	0 (95%CI 0.0-23.2)(0/14)	—	—
				PD-L1 ≥ 1%	46.4% (95%CI 27.5-66.1)(13/28)	—	—

^*^IC0: < 1% PD-L1-positive immune cells, IC1: ≥ 1% but < 5% PD–L1-positive immune cells, IC0/1: < 5% PD-L1-positive immune cells, IC2/3: ≥ 5% PD-L1-positive immune cells, IC1/2/3: ≥ 1% PD–L1-positive immune cells. OS: Overall survival, PFS: Progression free survival.

^[1]^ PD–1inhibitors: nivolumab and pembrolizumab.

^[2]^ PD–L1 inhibitors: atezolizumab and durvalumab.

### Patients with higher-ratio of PD-L1-positive cells responded better to PD-1/PD-L1 antibodies

We first tested whether patients with higher-ratio of PD-L1-positive cells responded better to PD-1/PD-L1 antibodies. Either of 1% and 5% was often used in the literature to classify patients into “positive” and “negative” groups, according to whether there are more than 1% or 5% PD-L1-positive cells in a patient.

**Figure 2:**
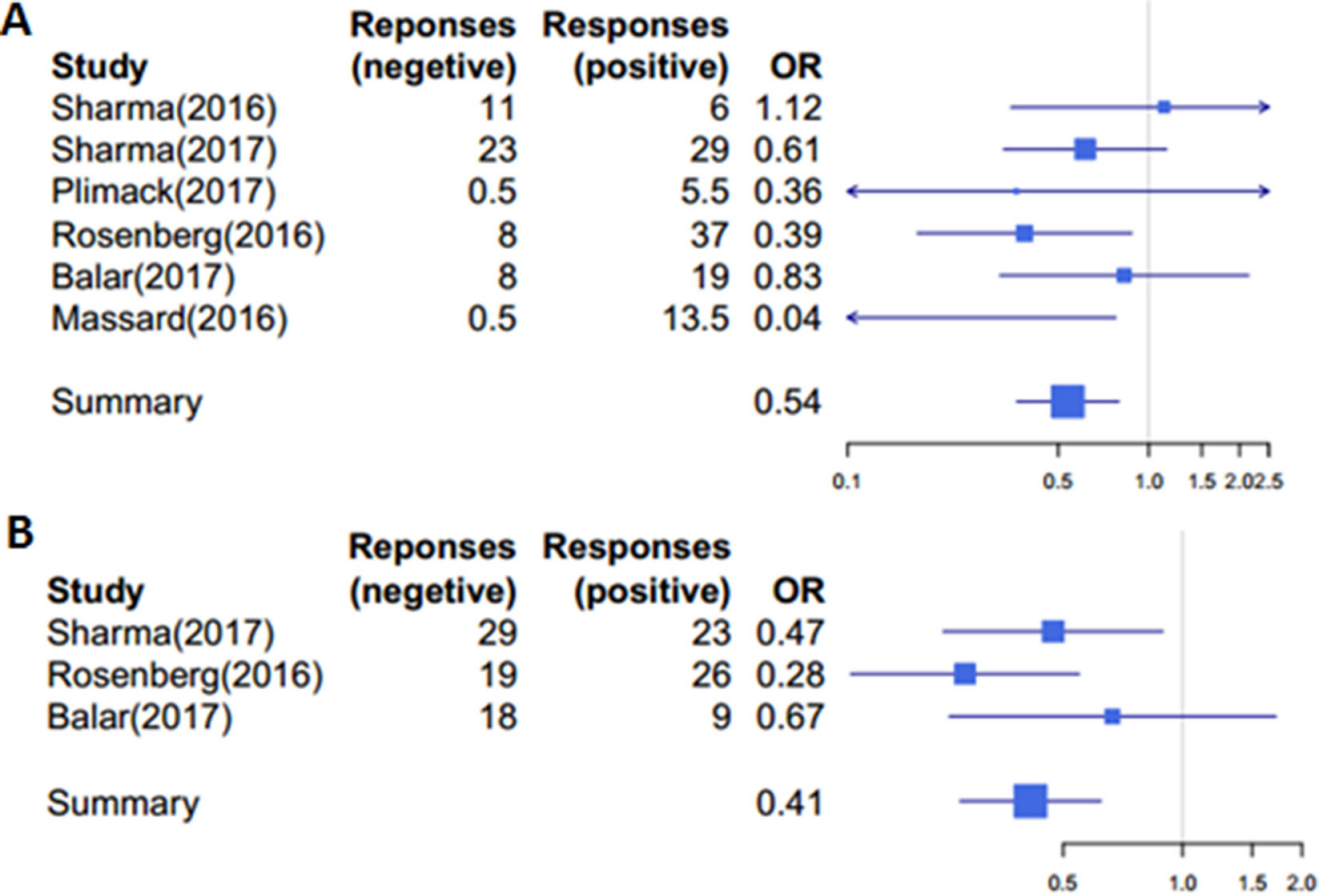
Patients with higher ratio of PD–L1-positive cells responded better to PD–1/PD–L1 antibodies (**A**) A cutoff of 1% was used to group patients into high (≥ 1%) and low (< 1%) subgroups, *P* = 0.004, ORs = 0.54, 95%CI: 0.39–0.85. (**B**) A cutoff of 5% was used to group patients into high (≥ 5%) and low (< 5%) subgroups, *P* = 3.78× 10−5, ORs = 0.41, 95%CI: 0.28–0.65. Responses: the number of patients achieved objective response; OR: the odds ratio of ORR with positive patients to negative ones.

Of all the studies, 50 of the 345 (14.50%) patients with PD-L1 ≥ 1% level achieved objective responses, while 109 of 483 (22.57%) patients with PD-L1 < 1% level achieved objective response, confirming that patients with higher-ratio of PD-L1-positive cells indeed responded better to PD-1/PD-L1 antibodies (*P* = 0.004, OR = 0.54,95%CI:0.39-0.85; see also Figure 2A).

We obtained similar results using 5% as the cutoff to divide patients into subgroups. Three out of the total six studies divided patients according to the 5% cutoff. In total, 50of 213 (23.47%) patients with PD-L1 ≥ 5% level achieved objective responses, as compared with 66 objective responses out of 481 (13.72%) patients with PD-L1 < 5% level; therefore patients with higher-ratio of PD-L1-positive cells responded better to PD-1/PD-L1 antibodies (*P* = 3.78, OR = 0.41,95%CI:0.28-0.65; Figure 2B).

### Patients with PD-L1 < 1% could respond better to PD-1 antibodies than to PD-L1 antibodies

**Figure 3:**
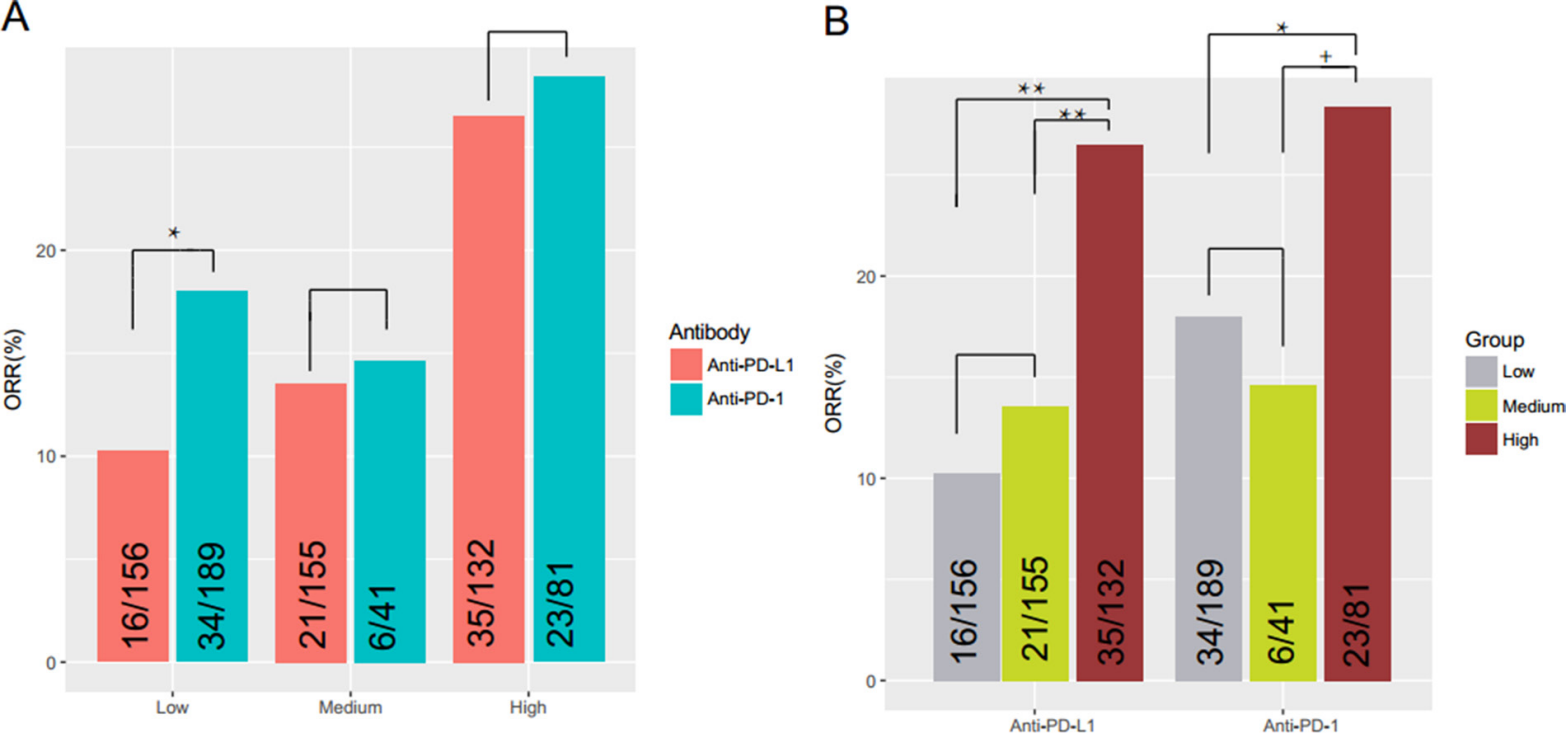
Effectiveness of PD–1/PD–L1 antibodies in patients with different PD–L1–positive cell ratios (**A**) No significant differences were found in the PD–L1 ≥ 1% but < 5% and PD–L1 ≥ 5% subgroups between PD–1 inhibitors and PD–L1 inhibitors (*P* = 0.80, OR = 1.09, 95%CI: 0.34-3.08; *P* = 0.87, OR = 1.09, 95%CI: 0.56–2.13, respectively). In PD–L1 < 1% group, patients had significantly better objective responses to PD–1 antibodies than to PD–L1 antibodies (*P* = 0.046, OR = 1.92, 95%CI: 0.98, 3.89). (**B**) There was significant difference between patients in the PD–L1 ≥ 5% subgroup responded better than PD–L1 < 1% and PD–L1 ≥ 1% but < 5% subgroups (*P* = 0.0003, OR = 0.45, 95%CI: 0.29–071; *P* = 0.0009, OR = 0.43, 95%CI: 0.25–0.73). No significant differences were found between the PD–L1 < 1% andPD–L1 ≥ 1%but < 5% subgroups (*P* = 0.90, OR = 1.06, 95%CI: 0.62–1.83).No significant differences were found in the Medium and High subgroups, but *P* value was close to 0.05 (*P* = 0.069, OR = 0.44, 95%CI: 0–1.077), which may because of very limited numbers of patients in the two subgroups. Low: PD–L1 expression < 1%; Medium: PD-L1 expression ≥ 1%but < 5%; High: PD–L1 expression ≥ 5%. +: 0.05 ≤ *P* < 0.10; ^*^:0.01 ≤ *P* < 0.05; ^**^:0.001 ≤ *P* < 0.01.

We next sought to compare the effectiveness of PD-1 and PD-L1 antibodies in patients with different PD-L1-positive cell ratios. In the literature, patients were often grouped into the following subgroups: PD-L1 < 1%, PD-L1 ≥ 1% but < 5%, and PD-L1 ≥ 5%; we referred them as to Low-PD-L1, Medium-PD-L1 and High-PD-L1 groups respectively in the following analysis. As shown in Figure 3A, we found no significant differences between the two types of antibodies in the Medium- and High-groups (*P* > 0.05, Fisher’s Exact Test). In some studies, patients of the two groups were often combined; again, we found no significant differences in the combined datasets in the responses to PD-1 and PD-L1 antibodies.

Surprisingly, we found in the Low-group, patients had significantly better objective responses to PD-1 antibodies than to PD-L1 antibodies (*P* = 0.046, OR = 1.92, 95%CI: 0.98, 3.89; Fisher’s Exact Test) (Figure 3A). It is known that PD-1 antibodies block the interaction between PD-1 with PD-L1 and PD-L2, while PD-L1 antibodies only block the interaction between PD-1 with PD-L1 [7, 23]; therefore it is very likely that PD-1 antibodies are more sensitive to lower ratio of PD-L1-possitive cells than to PD-L1 antibodies.

### 5% level of the PD-L1 positive cells should be used as the cutoff to distinguish PD-L1 positive patients from the negative ones

We also compared the responses of patients with different PD-L1-positive cell ratios to PD-1/PD-L1 antibodies. As shown in Figure 3, we found that patients in the High-group responded better than the other two groups (*P* = 0.0003, OR = 0.45, 95%CI: 0.29-071 for the High- versus Medium- comparison; *P* = 0.0009, OR = 0.43, 95%CI: 0.25-0.73 for the High- versus Low- comparison; Fisher’s Exact Test). However, patients in the Low- and Medium- groups responded similarly to both types of antibodies (*P* = 0.90, OR = 1.06, 95%CI: 0.62-1.83) (Figure 3B). These results suggested that a PD-L1-positive ratio of 5% or greater instead of 1% should be used as a threshold to define PD-L1 “positive” patients.

## DISCUSSION

In this study, we performed a meta-analysis on the effectiveness of anti-PD-1/PD-L1 antibodies in urothelial carcinoma patients with different ratios of PD-L1-positive cells. We first confirmed previous findings that patients with higher ratios of PD-L1-positive cells responded significantly better to both PD-1 and PD-L1 antibodies than those with lower ratios of PD-L1-positive cells.

We also checked whether patients responded better to one type of antibodies than the other. We found that in most cases, patients had similar ORRs between the two types of antibodies in the Medium-PD-L1 and High-PD-L1 groups. However, in patients with less than 1% PD-L1-positive cells (the Low-group), the objective response ratio was statistically better to PD-1 antibodies than to PD-L1 antibodies. This is likely that PD-1 antibodies are more sensitive to lower ratio of PD-L1-possitive cells, becausePD-1 antibodies block the interaction between PD-1 with PD-L1 and PD-L2, while PD-L1 antibodies only block the interaction between PD-1 with PD-L1 [7, 23]. However, due to limited numbers of patients involved in the studies, further clinical trials should be conducted in order to fully compare the effectiveness of all PD-1/PD-L1 antibodies in patients in the Low-group.

The definition of PD-L1 “positive” patients is not very clear so far [18]. Cutoffs of 1%, 5%, 10%have been used in many studies of urothelial cancer [21, 24], as in other cancers, such as NSCLC and melanoma [17, 25, 26]. For example, in the six studies analyzed, four chose 1% as the cutoff to define “positive” and “negative” patients. Our analysis indicates that patients in the Low-(PD-L1 < 1%) and Medium- (PD-L1 ≥ 1% but < 5%) groups had similar ORRs to PD-1/PD-L1 antibodies; medians OS and median PFS are also similar in the two groups. Conversely, the ORRs to the antibodies in the High-group (PD-L1 ≥ 5%) were significantly better than the other two groups. These results suggested a ratio of PD-L1-positive cells of 5% and greater should be used as the cutoff to define PD-L1-“positive” patients in urothelial cancer. The 5% cutoff is also used in other cancers [27]. However, recently Skala and Farajre commended a much higher cutoff (i.e. 50%) for urothelial cancer [28, 29]. Due to limited data so far, we could not rule out the possibility that a higher ratio (such as 10% or even 50%) should be used.

In conclusion, PD-L1 positive patients of urothelial cancer response more than negative ones, and we suggest 5% or greater as the cutoff to distinguish them. PD-1 antibodies is better than PD-L1 antibodies in PD-L1 < 1% subgroup with urothelial carcinomas patients. Further clinical trials need to be performed to establish the position of immunotherapy in urothelial cancer.

## MATERIALS AND METHODS

### Literatures search

We performed a systematic review search of literatures in Pubmed, Embase, clinical trials.gov and American Society of Clinical Oncology (ASCO) meeting abstracts for relevant articles published in English. All the articles were published before July, 2017. We used the following keywords to search for the articles: “PD-1”, “PD-L1” and “urothelial carcinoma”, “urothelial cancer”, “urothelial tumor” combined with “nivolumab”, “atezolizumab”, “pembrolizumab”, “avelumab”, “durvalumab”.

### Selection criteria

The articles must satisfy the following conditions: first, all patients suffering from advanced and metastatic urothelial carcinoma were treated with PD-1/PD-L1 antibodies. Second, certain types of raw data should be available in the original publications in order for us to combine results from multiple studies, including the total numbers of patients treated by PD-1/PD-L1 antibodies, the numbers of patients that responded to the treatment (note: either directly available or could be calculated from objective response rates (ORRs)), and the percentage of PD-L1-positive cells in the tumor microenvironment of these patients. In cases where the exact PD-L1-positive levels were unknown, patients should be grouped into sub-groups according to their rates of PD-L1-positive levels and the grouping criteria should be available.

### Data extraction

The data extracted from these articles was as below: the first author, the year of the article, the trial name, the antibody name, the different levels of PD-L1 expression and ORR (objective response rate), media progression-free survival (PFS), media overall survival (OS).

### Outcome for analysis

The outcomes were ORRs and ORs of different PD-L1 levels in urothelial carcinoma patients by different antibodies.

### Statistical analysis

All analyses were performed using R 3.4.1 (https://www.r-project.org) and R Studio (https://www.rstudio.com). Patients were grouped into subgroups according to the percentages of PD-L1-positive cells. The ORR value was calculated for each subgroup. Between-subgroup comparisons were performed by one-tailed Fisher-test using the Fisher. test (matrix) function in R; Odds ratio (OR), its 95% confidence interval (95%CI) and the *P*-value were recorded for each comparison. It was to consider statistically significant if *P* < 0.05.
